# Investigation of Health Effects According to the Exposure of Low Concentration Arsenic Contaminated Ground Water

**DOI:** 10.3390/ijerph14121461

**Published:** 2017-11-27

**Authors:** Young-seoub Hong, Byeong-jin Ye, Yu-mi Kim, Byoung-gwon Kim, Gyeong-hui Kang, Jeong-jin Kim, Ki-hoon Song, Young-hun Kim, Jeong-wook Seo

**Affiliations:** 1 Department of Preventive Medicin, Dong-A University, Busan 49201, Korea; yshong@dau.ac.kr (Y.H.); kimyumi@dau.ac.kr (Y.K.); medikim@dau.ac.kr (B.K.); 2 Environmental Health Center, Dong-A University, Busan 49201, Korea; 3 Gimhae Clinic Occupational Health Center, Inje University, Gimhae 50969, Korea; ong94@hanmail.net; 4 Haman Community Healthcare center, Haman 50461, Korea; angel3395@korea.kr; 5 Department of Earth and Environmental Science, Andong National University, Andong 36729, Korea; jjkim@andong.ac.kr; 6 Department of Dermatology, Dong-A University, Busan 49201, Korea; khsong@dau.ac.kr; 7 Department of Environmental Engineering, Andong National University, Andong 36729, Korea; youngkim@andong.ac.kr

**Keywords:** arsenic, biomonitoring, health effects

## Abstract

Recent epidemiological studies have reported adverse health effects, including skin cancer, due to low concentrations of arsenic via drinking water. We conducted a study to assess whether low arsenic contaminated ground water affected health of the residents who consumed it. For precise biomonitoring results, the inorganic (trivalent arsenite (As III) and pentavalent arsenate (As V)) and organic forms (monomethylarsonate (MMA) and dimethylarsinate (DMA)) of arsenic were separately quantified by combining high-performance liquid chromatography and inductively coupled plasma mass spectroscopy from urine samples. In conclusion, urinary As III, As V, MMA, and hair arsenic concentrations were significantly higher in residents who consumed arsenic contaminated ground water than control participants who consumed tap water. But, most health screening results did not show a statistically significant difference between exposed and control subjects. We presume that the elevated arsenic concentrations may not be sufficient to cause detectable health effects. Consumption of arsenic contaminated ground water could result in elevated urinary organic and inorganic arsenic concentrations. We recommend immediate discontinuation of ground water supply in this area for the safety of the residents.

## 1. Introduction

Arsenic is present in the form of inorganic arsenic and organic arsenic in ecological environments. The toxicity of arsenic is different according to the type, and inorganic arsenic is more toxic than organic arsenic, of these, trivalent arsenite is more toxic than pentavalent arsenate [1]. Inorganic arsenic is mainly derived from the environment, and organic arsenic is mainly consumed as food. Therefore, in the risk assessment, it is necessary to distinguish between inorganic arsenic and organic arsenic.

Arsenic exposure via drinking water is a very important public health concern worldwide. The severity of the health effects of arsenic in drinking water is constantly being reevaluated by various government agencies. Hyperkeratosis, hyperpigmentation, and hypopigmentation [2] are representative skin symptoms that are the first to be manifested by chronic arsenic exposure and are also used as clinical indicators of arsenic exposure. In addition, exposure to arsenic in drinking water has been associated with respiratory, neurological, and diabetic mellitus. It has been reported that chronic respiratory inflammation are caused by arsenic-induced respiratory diseases [3], and peripheral neuropathy, encephalopathy, and polyneuropathy are caused by nervous system diseases [4]. An epidemiologic report on the risk of miscarriage and preterm birth in chronic exposure to arsenic during pregnancy [5], and the report of congenital anomalies [6] suggest that exposure to arsenic may cause reproductive and developmental abnormalities [7].

Furthermore, various harmful health effects due to exposure to low concentrations of inorganic arsenic have been reported in many studies [8,9,10,11]. In a recent epidemiological study, various health effects, including skin cancer, have been reported to be caused by exposure to low concentrations of arsenic (10 µg/L) via drinking water [12]. Some studies have also shown that well water samples containing arsenic concentrations of >1.0 µg/L were associated with skin cancer [13].

Therefore, the United States Environmental Protection Agency, World Health Organization, and the Food and Drug Administration have all lowed the international cut off for total arsenic concentration in drinking water from 50 µg/L to 10 µg/L [14,15]. In accordance with this global trend, Korea has also decreased the cut off from 50 µg/L to 10 µg/L in the 2007 [16].

All local governments under the Korean system are required to conduct water quality tests annually according to the law. As a result of the water quality test, some area residents, including children, were found to have taken arsenic contaminated drinking water that exceeds the maximum allowable criteria (10 µg/L). Moreover, owing to poor knowledge of the toxicity of the arsenic, communities have excessive anxiety of arsenic toxicity health risks. It has been raised the need of a health impact assessment and an epidemiological study by local residents and non-governmental organization.

The aim of this study was to investigate and evaluate arsenic species concentration and the health effects that are associated with short-term (within one year) arsenic exposure via contaminated drinking water. The secondary aim was to establish prevention strategies for arsenic related diseases by determining the route of arsenic exposure, and to disseminate relevant scientific information within communities that have expressed anxiety towards the health effects of arsenic exposure.

## 2. Materials and Methods

### 2.1. Participants

**Figure 1:**
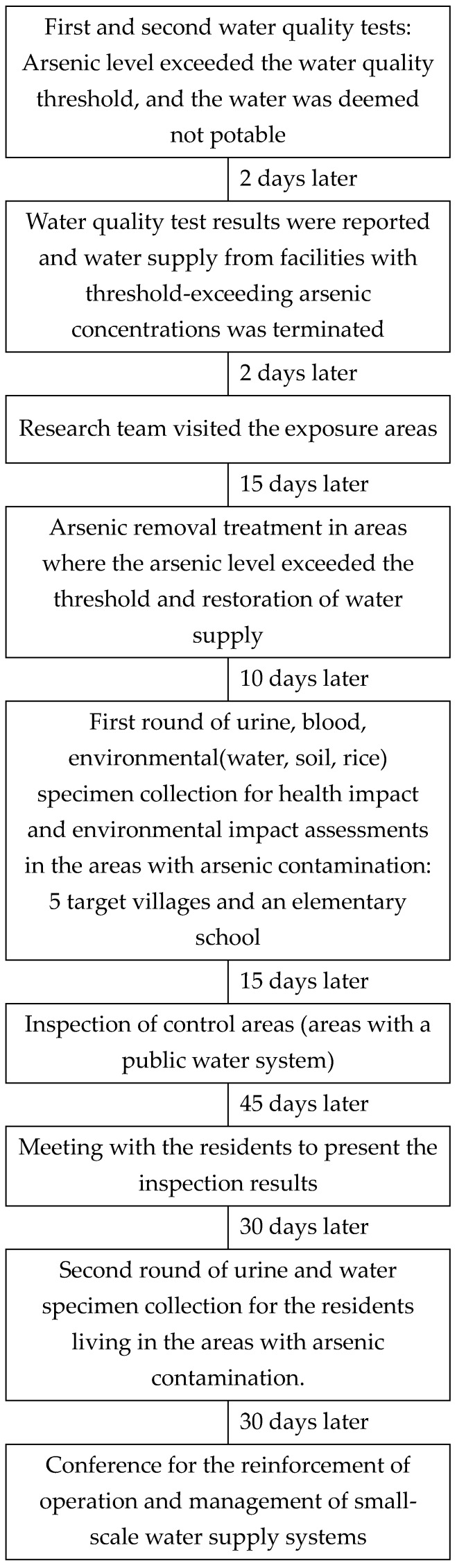
Survey flow.

The overall survey follow according the passage of time is shown in Figure 1.

Among the water quality tests that were conducted on small-scale water supply systems in Korea (temporary water supply, ground water), we used data for five facilities with arsenic concentrations exceeding the allowable threshold in our analysis. These five water treatment facilities supplied water to five villages and one elementary school. The residents and elementary school students who used the water from these facilities were enrolled (*n* = 144). Residents of areas with similar (i) living conditions, (ii) age distribution, and (iii) population size as the exposure areas, wherein (iv) cooperation with residents would be smooth, and (v) water was supplied from a public water supply system and not from a temporary system were selected as control participants (*n* = 65).

To minimize selection bias, we conducted a complete enumeration survey. Prior to the study, all households were visited, and the residents were informed about arsenic exposure in drinking water. Those residents who agreed to participate in the study were included in the health impact assessment surveys. This study had a community-based intervention design.

### 2.2. Sample Collection

According to the registration data, the population of the five target villages comprised 132 households and 247 residents. Thus, a total of 99 adults, including 83 residents (33.6%) and 16 individuals staying in the villages for their business, participated in the health impact survey. Additionally, 45 children aged below 12 years attending the elementary schools in the exposure areas were surveyed in the same manner as the adult participants. The health impact assessment included a survey questionnaire and collection of blood, urine, and hair specimens. Blood specimens were collected into BD Vacutainer tubes, in which an anticoagulant was added; the tubes were placed on a roll mixer to prevent coagulation and then stored in a deep freezer at –80 °C until analysis. After the participants were instructed regarding the urine collection method to prevent contamination, spot urine specimens were collected, divided into several tubes, and stored in a deep freezer at –80 °C until analysis. Hair specimens were stored after being secured in hair specimen envelopes.

The environmental impact assessment comprised water, soil, and crop analyses. In the target villages, water that was pumped out from the groundwater well was disinfected in a storage tank and distributed to each house. Drinking water samples were collected from the source well of each village. In general, two samples (before and after application of an arsenic filter) were collected from each groundwater well. The water sample was collected in a prewashed plastic sampling bottle after pumping approximately 40 L of water into the pipeline. The water samples were stored in an ice box until laboratory analysis. Surface soil (0−10 cm depth) was collected from farmland in the studied area with a prewashed hand auger and transferred to plastic bags. The soil samples were air-dried for five days and sieved with a 100-mesh sieve for acid digestion. Samples of crops such as rice, red pepper, and beans were collected from the same farmland area around the groundwater wells as the soil sample, and were washed with distilled water.

### 2.3. Metal Analysis 

Urine specimens were filtered using a 0.22 µm filter to remove impurities and appropriately diluted in distilled water. Speciation was carried out by using a phosphate buffer via a high-performance liquid chromatography (HPLC) system (Agilent Technologies 1260, Santa Clara, CA, USA), on which a Hamilton PRP X-100 column was mounted. The inorganic form (trivalent arsenite (As III) and pentavalent arsenate (As V) and organic forms (monomethylarsonate (MMA) and dimethylarsinate (DMA)) of arsenic were separately quantified by combining HPLC and inductively coupled plasma-mass spectroscopy (ICP-MS) (Agilent Technologies 7700 series, Santa Clara, CA, USA). Calibration curves were constructed based on the standards including As III, As V, DMA, and MMA, and the accuracy of the analysis was confirmed using two types of standard reference material (SRM, NIST SRM 2669 & NIES No. 18).

Arsenic and metals (Cd, Cu, Hg, Pb, Cr, Zn, Ni) in water, soil, and rice were analyzed for environmental exposure assessment. The water samples were acidified with a few drops of nitric acid and analyzed via ICP-MS, which refers to American Public Health Association, the American Water Works Association, and the Water Environment Federation standard method (Part 3125) [17], and Environmental Protection Agency method (EPA) method (Method 200.8) [18]. 

An EPA method (Method 3051) [19] was used for the extraction of metals from the soil samples. The air-dried soil samples (0.5 g) were placed in a pre-acid washed Teflon tube and 9.5 mL of nitric acid was added. The Teflon tube was installed in a microwave system wherein the extraction took place for 15 min. The extracted solution was diluted with 1.0% nitric acid and analyzed with ICP atomic emission spectroscopy (Agilent Technologies 720 series, Santa Clara, CA, USA). 

Rice samples were polished and the edible portions of the other crops were freeze-dried. The dried samples were ground with a mortar, and approximately 0.5 g of the ground crop sample was digested using 9.5 mL of nitric acid in a microwave digestion equipment. The prepared sample solution was diluted with water and analyzed with ICP-MS.

### 2.4. Health Examination

We conducted a health examination to evaluate the health effects of the arsenic exposure. The health examination test included complete blood cell count, liver function test, renal function test, chest radiography, and electrocardiogram as a diagnostic test. Tumor markers, such as alpha fetoprotein, carcinoembryonic antigen, carbohydrate antigen 19-9, cancer antigen 125, prostate specific antigen, and cytokeratin-19 fragments were used as tumor markers for liver, colon, pancreas, ovary, prostate, and lung respectively.

### 2.5. Statistical Analysis

After speciation, the distribution of arsenic concentrations in urine and hair were found to be skewed (skewness > 0). Hence, the geometric mean (GM) and 95% confidence interval (95% CI) that were adjusted for general characteristics were computed. Arsenic concentration was compared between the adult exposure and control groups, and between the child exposure group and adult control group. In the exposed group, the creatinine level was not more than 300–3000 mg/L. In the control group, creatinine analysis was not performed. To compare the two groups, we used the value before creatinine correction. The GM (95% CI) for main source of drinking water was computed for the exposure group. Additionally, changes in arsenic concentration between the first and second assessments were examined. All of the statistical tests were conducted at a significance level of 5% using SAS (Version 9.4, SAS Institute, Cary, NC, USA).

### 2.6 Ethics

The protocol of this study was reviewed and approved by the Institutional Review Board of the Dong-A university hospital (IRB No. 13-010). Written informed consents were provided by all of the participants.

## 3. Results

### 3.1. Participant Characteristics

Table 1 shows the demographic and lifestyle characteristics of the exposure and control groups. There was a significant difference in sex, age, education level, job, duration of residence, and drinking water source between the two adult groups.

### 3.2. Comparison of Urinary Arsenic Concentrations

The GM (95% CI) of the urinary arsenic concentrations were compared after adjusting for demographic and lifestyle characteristics in addition to the geographic characteristics that distinguished the adult exposure and control groups. The results showed that the inorganic arsenic concentration was significantly higher in the exposure group (2.31 (1.62–3.31) µg/L) than in the control group (0.94 (0.61–1.44) µg/L, *p* < 0.001). Specifically, a significant difference was found in terms of As V (exposure group: 0.59 (0.37–0.91) µg/L; control group: 0.19 (0.11–0.32) µg/L; *p* < 0.001), but not As III (*p* = 0.239). The organic arsenic concentration was also higher in the exposure group than in the control group (exposure group: 63.54 (48.50–83.25) µg/L; control group: 42.53 (30.85–58.63) µg/L; *p* = 0.007). A significant between-group difference was found in terms of both DMA (exposure group: 58.88 (44.64–77.65) µg/L; control group: 39.68 (28.56–55.14) µg/L; *p* = 0.010); and, MMA (exposure group: 3.89 (2.86–5.29) µg/L, control group: 2.15 (1.49–3.10) µg/L; *p* = 0.001). The adjusted hair arsenic level of the exposure group was 0.19 (0.15–0.24) ppm, which was significantly higher than that of the control group (0.12 (0.09–0.15) ppm; *p* < 0.001). Urinary trivalent and pentavalent inorganic arsenic concentrations among adult residents were 1.04 µg/L and 0.59 µg/L, respectively, which were significantly higher than those among control site residents (0.71 µg/L and 0.19 µg/L, respectively). Moreover, the pentavalent arsenic concentration of the children in this region was 1.29 µg/L, which was significantly higher than that among control site residents (0.37 µg/L) (Table 2). However, the urinary inorganic arsenic concentration in the participants was low (≤10 µg/L) and did not exceed the international criteria (10 µg/L).

The results of the analysis conducted to compare the child and adult exposure groups showed that the GM (95% CI) of inorganic arsenic was 2.03 (1.75–2.37) µg/L in the child exposure group, which was significantly higher than the 1.18 (0.93–1.49) µg/L that was observed in the control group. Particularly, a marked difference was found in terms of As V (exposure group: 1.29 (1.16–1.44) µg/L; control group: 0.37 (0.29–0.47) µg/L; *p* < 0.001). In contrast, the organic arsenic concentrations were significantly lower in the child exposure group than in the control group (exposure group: 40.19 (32.40–49.85) µg/L; *p* = 0.046). The arsenic concentration in hair was significantly higher in the child exposure group (0.15 (0.13–0.17) ppm) than in the control group (0.10 (0.09–0.11) ppm; *p* < 0.001) (Table 2).

### 3.3. Urinary Arsenic Concentration According to Drinking Water Source

The adjusted GM (95% CI) of inorganic arsenic was higher in the adult exposure group that consumed contaminated groundwater, at 2.87 (1.62–5.10) µg/L, when compared to the group that consumed tap water, at 1.82 (0.84–3.95) µg/L, but the difference was not statistically significant (*p* = 0.114). In particular, the concentration of As V was 0.71 (0.31–1.65) µg/L in contaminated groundwater, which was approximately three times higher than that in tap water (0.26 (0.08–0.80) µg/L) and showed a statistically significant difference (*p* < 0.001). The concentration of As III in contaminated groundwater 1.79 (0.61–5.29) µg/L was more than twice that in tap water (0.75 (0.17–3.24) µg/L), but the difference was not statistically significant (*p* = 0.109). The adjusted concentration of organic arsenic in contaminated groundwater was 68.53 (44.93–104.52) µg/L, which was higher than that in tap water (43.85 (24.35–78.94) µg/L), but the difference was not statistically significant (*p* = 0.105). Likewise, the concentration of arsenic in hair did not show a statistically significant difference (*p* = 0.321).

The analysis that was conducted to compare arsenic concentrations of the child exposure group according to source of drinking water revealed that the inorganic arsenic concentration was 2.09 (1.73–2.52) µg/L in those for whom the main source of drinking water was contaminated groundwater, which was somewhat higher than that in residents who consumed tap water, at 1.84 (1.46–2.32) µg/L, but without statistical significance (*p* = 0.494). Particularly, the concentration of As III was 0.46 (0.28–0.77) µg/L in contaminated groundwater and 0.16 (0.06–0.41) µg/L in tap water, indicating that the concentration of the former was more than twice that of the latter, with borderline significance (*p* = 0.053). Organic arsenic concentration did not show a statistically significant difference between these groups (*p* = 0.424). Similarly, there was no statistically significant difference in the concentration of arsenic in hair (*p* = 0.484) (Table 3).

### 3.4. Changes in Urinary Arsenic Concentration According to Timing of Tests 

The second collection of urinary specimens was performed approximately three months after the first health impact survey was conducted in the areas exposed to arsenic. Changes in concentration were examined (Table 4).

The number of individuals in the adult exposure group whose urinary arsenic level was analyzed in the first assessment was 97, and 74 of these participants were followed up in the second assessment. For all of the urinary arsenic species, including inorganic arsenic, the concentration was lower in the second assessment than that in the first. The concentration of inorganic arsenic decreased by 2.19 µg/L ± 1.90 µg/L, and it was below the limit of detection in most of the participants (*p* < 0.001). Specifically, the concentration of As V decreased by 0.93 µg/L ± 0.90 µg/L and As III by 1.27 µg/L ± 1.59 µg/L, with both values demonstrating statistically significant differences (*p* < 0.001). Likewise, the concentration of organic arsenic decreased by 12.54 µg/L ± 69.97 µg/L (*p* = 0.127). In particular, the concentration of MMA decreased by 1.58 µg/L ± 3.73 µg/L, showing a statistically significant difference (*p* < 0.001). The arsenic concentration decreased from the first assessment to the second in 98.7% of participants, with the exception of 1.4% in whom the inorganic arsenic concentration remained the same, and the mean decrease was 2.22 µg/L ± 1.89 µg/L.

The number of individuals in the child exposure group whose urinary arsenic level was examined during the first assessment was 45, and all of them were followed up during the second assessment. The mean difference in organic arsenic concentration between the first and second assessment was 5.93 µg/L ± 45.29 µg/L, indicating a slight increase, but statistical significance was not achieved (*p* = 0.385). The concentration of inorganic arsenic reduced by 1.94 µg/L ± 1.21 µg/L, and was below the limit of detection in almost all of the participants (*p* < 0.001). Specifically, the concentration of As V decreased by 1.17 µg/L ± 0.65 µg/L and As III by 0.76 µg/L ± 1.01 µg/L, and the decrease was statistically significant (*p* < 0.001). The concentration of inorganic arsenic was confirmed to have decreased from the first assessment to the second in all participants.

### 3.5. Comparison of the Diagnostic Test Results among Adult Participants

Most health screening results did not show a statistically significant difference between groups, but systolic blood pressure, white blood cell count, and carbohydrate antigen 19-9 (marker of pancreatic cancer) level showed a statistically significant difference. However, systolic blood pressure and white blood cell count were higher in the control group, while carbohydrate antigen 19-9 level was higher in the exposure group. Nevertheless, most of the results fell within the normal range (Table 5).

### 3.6. Changes in Arsenic Concentration in the Water Supply in the Exposure Areas

The concentration of arsenic in the drinking water of the studied area was much higher than the Korean drinking water standard (0.010 mg/L) at 0.018–0.104 mg/L. The data obtained from the second screening showed a very similar range: 0.016–0.097 mg/L. However, the local government installed a drinking water filter for arsenic soon after they recognized the water contamination. Subsequently, the concentration of arsenic decreased to below 0.01 mg/L (Table 6).

### 3.7. Arsenic Concentration in the Soil and Crops Surrounding the Exposure Areas

The arsenic concentration in farmland soil ranged 5.27–16.37 mg/kg (Table 7), which was below the Korean soil environmental limit of 25 mg/kg. The arsenic concentration in rice samples ranged 0.03–0.212 mg/kg, which was below the Korean food environmental limit of 0.2 mg/kg.

## 4. Discussion

The present environmental impact assessment survey showed that the concentration of arsenic in the drinking water of the studied area ranged 18–104 µg/L, which was much higher than the Korean drinking water standard of 10 µg/L. The data obtained at the second screening stage showed a very similar trend: 16–97 µg/L. This indicates that water containing arsenic concentrations above the standard value has been supplied as drinking water for a few hundred local residents. Based on the geographic characteristic of the studied area, we presume that the contaminated water has been supplied for a few years after the small-scale water supply system has been installed; however, we do not have sufficient data to confirm this. Nonetheless, the local government installed a drinking water filter for arsenic soon after they recognized the water contamination. Subsequently, the concentration of arsenic related decreased to below 10 µg/L. The current study was initiated >2 months after the filter installation. 

Generally, arsenic toxicity is caused by the exposure to high levels of arsenic (>100 µg/L), and has shown a significant association with skin cancer, lung cancer, bladder cancer, and non-carcinogenic effects [20,21,22,23]. The non-carcinogenic effect has been reported to include human effects that are caused by ingestion of inorganic arsenic, including mucous membrane stimulation and skin lesions, peripheral neurotoxicity, keratosis, neuromuscular abnormalities, and vascular abnormalities [24].

Brown et al. [25] suggested the lifetime risk of developing skin cancer was 1.3/1000 for males and 0.6/1000 for females per microgram of arsenic per day in Taiwan. Moreover, the regression analysis showed that the cancer mortality rate (per 100,000) for bladder, kidney, lung, and liver cancer significantly increased per microgram increase in arsenic exposure via drinking water in a linear dose-response. Abernathy et al. [26] suggested the NOAEL 0.8 µg/kg/day, LOAEL 14 µg/kg/day using the Tseng et al. [27] and Tseng model [28]. In the United States of America (USA), the estimated lifetime risk of dying from cancer due to arsenic exposure via drinking water (1.6 L/day) was 1/1000 per 2.5 microgram of arsenic exposure per day [29]. Therefore, the possibility of disease is related to the amount of exposure. When considering the elevated arsenic concentrations, it may not be sufficient to cause detectable health effects in a period of less than one year, however, lifetime assessment should be conducted in future study. We presume that the elevated arsenic concentrations may not be sufficient to cause detectable health effects in a period of less than one year, however, human exposure of arsenic could occur via other chemical exposure pathways, including ingestion of contaminated residential soil, inhalation of soil dust, dermal contact to the residential soil [30], and ingestion of contaminated agricultural foods [31]. Therefore, it will be necessary to assess the health effects of multiple exposures when considering these factors in the future.

One of the limitations of this study was that we did not select a child control group. It was difficult to obtain control children who met the conditions to living condition, age distribution, and time and budget constraints. Although the adult control group was not satisfied as a case-control in comparison with the children exposure group, it was used as a reference to compare the concentration of inorganic arsenic that was exposed to contaminated water.

Our community-based health survey showed that the urinary organic and inorganic arsenic concentrations among residents were significantly higher than those of control site residents; moreover, the consumption of contaminated drinking water was found to urinary arsenic concentration in residents. When considering that the current study was initiated >2 months after the installation of the filter by the local government, and that the half-life of inorganic arsenic in the body is approximately 2–4 days, the level of arsenic exposure among residents in the past was higher than that recorded in the present study [32].

Recent reports have shown that exposure to low concentrations of arsenic could result in high blood pressure, obesity, high triglyceride, hyperglycemia, metabolic syndrome, anemia, and bone damage [33,34,35,36]. 

We evaluated the carcinogenic and non-carcinogenic effects arsenic exposure; however, we did not find any carcinogenic or obvious clinical abnormalities among exposure residents when compared with the control subjects. 

In this study, we applied the tumor marker test to evaluate the carcinogenicity of arsenic, although it is a nonspecific test for cancer screening. We selected tumor marker liver, colon, pancreas, ovary, prostate, and lung that carcinogenicity may be associated with the inorganic arsenic exposure. As a result of analysis, one subject who exceeded the reference value in Carbohydrate antigen 19-9 was found, but there was no clinical relevance according to the exposure of arsenic through close examination. All of the subjects were within normal range and there was no statistically significant difference between the exposure and control group. In general, this clinical tumor marker test is more applicable to follow up cancer management than cancer screening. Therefore, it is considered to be worthy of a reference test rather than a detection of cancer development due to arsenic exposure.

## 5. Conclusions

We presume that the elevated arsenic concentrations may not be sufficient to cause detectable health effects. Moreover, we analyzed these subjects for relatively short period. We also found that the consumption of arsenic contaminated groundwater could elevate urinary organic and inorganic arsenic concentrations. Therefore, the consumption of arsenic contaminated groundwater poses a health threat to the residents of the study area. We recommend immediate discontinuation of groundwater supply in this area for the safety of the residents. Nonetheless, urinary inorganic arsenic level was found to decrease dramatically overtime. This study has several limitations. First, it was difficult to accurately compare arsenic exposure concentrations before and after the introduction of the arsenic filtration system, since this study was conducted after the arsenic filtration systems were installed. Second, we did not perform the urinary creatinine normalization of arsenic in the comparison between exposed and control group. Nevertheless, the findings of this survey are important for policy making in Korea. This is the first epidemiological survey of arsenic contamination of drinking water in Korea. Moreover, this survey could bring about cooperation among community residents, non-governmental organization, and the local government without the support of government. We believe these findings could serve as important evidence for the management of exposure to arsenic-contaminated groundwater.

## Figures and Tables

**Table 1:** Participant characteristics.

Characteristics		Adult		*p*-Value	Child Exposure (Age ≤ 12)
		Exposure	Control		
Total		99 (60.4)	65 (39.6)		45
Sex	Male	35 (35.4)	12 (18.5)	0.019	24 (53.3)
	Female	64 (64.6)	53 (81.5)		21 (46.7)
Age (year)	Mean ± Standard deviation	62.87 ± 15.29	66.58 ± 8.03	0.073	7.69 ± 2.44
	19–49	15 (15.2)	1 (1.5)	0.001	
	50–59	24 (24.2)	10 (15.4)		
	60–69	20 (20.2)	30 (46.2)		
	≥70	40 (40.4)	24 (36.9)		
Education level (year)	Less than elementary school (<6)	23 (23.2)	15 (23.8)	0.012	
	Elementary school (6–9)	18 (18.2)	19 (30.2)		
	Middle school (9–12)	16 (16.2)	17 (27.0)		
	High school and more (≥13)	42 (42.4)	12 (19.0)		
Smoking status	Current-smoker	11 (11.1)	2 (3.1)	0.173	
	Former-smoker	15 (15.2)	10 (15.4)		
	Never-smoker	73 (73.7)	53 (81.5)		
Drinking status	Current-drinking	52 (52.5)	32 (49.2)	0.085	
	Former-drinking	13 (13.1)	17 (26.2)		
	Never-drinking	34 (34.3)	16 (24.6)		
Job	Skilled agricultural workers	33 (33.3)	49 (75.4)	<0.001	
	The others	66 (66.7)	16 (24.6)		
Duration of residence (year) (missing *n* = 8 in exp) (missing *n* = 8 in ctrl)	Mean ± Standard deviation	37.05 ± 24.69	41.07 ± 19.20	0.298	
	≤20	34 (37.4)	10 (17.5)	0.005	
	21–50	23 (25.3)	28 (49.1)		
	≥51	34 (37.4)	19 (33.3)		
Drinking water	Tap water	16 (16.2)	60 (92.3)	<0.001	10 (22.2)
	Mineral water	6 (6.1)	0 (0.0)		0 (0.0)
	Contaminated groundwater	77 (77.8)	5 (7.7)		35 (77.8)

**Table 2:** Comparison of arsenic concentrations.

Arsenic Species in Urine and Hair				GM (95% CI)			*p*-Value ^1^	*p*-Value ^2^
				Exposure		Control (*n* = 64)		
				Adult (*n* = 97)	Child (*n* = 45)			
Crude	Urine (µg/L)	Organic As	DMA	57.445 (49.289–66.949)	37.160 (29.820–46.305)	51.179 (41.652–62.884)	0.363	0.039
			MMA	3.620 (3.030–4.325)	2.361 (1.741–3.203)	2.549 (2.037–3.188)	0.015	0.680
			Subtotal	61.781 (53.156–71.804)	40.190 (32.405–49.846)	54.396 (44.463–66.548)	0.307	0.046
		Inorganic As	As III	0.778 (0.556–1.087)	0.366 (0.233–0.576)	0.586 (0.419–0.819)	0.256	0.090
			As V	0.736 (0.566–0.956)	1.294 (1.160–1.442)	0.366 (0.286–0.467)	<0.001	<0.001
			Subtotal	2.144 (1.752–2.624)	2.034 (1.748–2.366)	1.176 (0.928–1.491)	<0.001	<0.001
		Total		64.561 (55.617–74.942)		56.052 (45.904–68.444)	0.253	0.065
	Hair (ppm)Exposure *n* = 98Control *n* = 65			0.161 (0.138–0.186)		0.097 (0.085–0.111)	<0.001	<0.001
Adjusted	Urine (µg/L)	Organic As	DMA	58.880 (44.644–77.654)		39.682 (28.559–55.136)	0.010	
			MMA	3.890 (2.861–5.289)		2.149 (1.492–3.096)	0.001	
			Subtotal	63.543 (48.500–83.252)		42.527 (30.848–58.627)	0.007	
		Inorganic As	As III	1.035 (0.573–1.870)		0.706 (0.350–1.426)	0.239	
			As V	0.585 (0.374–0.915)		0.190 (0.112–0.323)	<0.001	
			Subtotal	2.314 (1.616–3.314)		0.937 (0.611–1.435)	<0.001	
		Total		66.558 (50.954–86.940)		43.920 (31.973–60.332)	0.005	
	Hair (ppm)			0.187 (0.149–0.226)		0.117 (0.089–0.154)	<0.001	

^1^ Comparison between the adult exposure and adult control group; ^2^ Comparison between the child exposure and adult control group; GM (95% CI): geometric means (95% confidence limit); Organic arsenic: DMA + MMA; Inorganic arsenic: As III + As V; Total urine arsenic: DMA + MMA + As III + As V; Limit of detection: As III 0.176; As V 0.135; DMA 0.121; MMA 0.198; Adjusted for sex, age, education level, smoking status, drinking status, job, duration of residence, drinking water.

**Table 3:** Comparison of arsenic concentrations between the adult and child exposure groups according to source of drinking water.

**Arsenic Species in Urine and Hair**				**Adjusted GM (95% CI)**		***p***-**Value**
				**Contaminated Ground Water (*n* = 66)**	**Tap Water (*n* = 14)**	
Adult	Urine (µg/L)	Organic As	DMA	62.487 (40.451–96.527)	43.845 (24.353–78.939)	0.105
			MMA	5.532 (3.762–8.134)	3.999 (2.375–6.735)	0.094
			Subtotal	68.531 (44.933–104.521)	48.222 (27.250–85.332)	0.098
		Inorganic As	As III	1.791 (0.606–5.294)	0.748 (0.173–3.238)	0.109
			As V	0.710 (0.307–1.646)	0.256 (0.082–0.797)	0.017
			Subtotal	2.871 (1.617–5.097)	1.819 (0.837–3.953)	0.114
		Total		72.118 (47.556–109.364)	50.665 (28.853–88.965)	0.092
	Hair (ppm)			0.157 (0.102–0.241)	0.127 (0.071–0.226)	0.321
**Arsenic species in urine and hair**				**GM (95% CI)**		***p***-**Value**
				**Contaminated Ground Water (*n* = 35)**	**Tap Water (*n* = 10)**	
Child	Urine (µg/L)	Organic As	DMA	35.383 (27.216–46.000)	44.111 (28.452–68.388)	0.407
			MMA	2.178 (1.482–3.199)	3.135 (2.310–4.255)	0.125
			Subtotal	38.249 (29.562–49.490)	47.792 (31.428–72.675)	0.392
		Inorganic As	As III	0.462 (0.276–0.773)	0.163 (0.064–0.413)	0.053
			As V	1.256 (1.102–1.432)	1.433 (1.172–1.752)	0.316
			Subtotal	2.091 (1.734–2.522)	1.845 (1.464–2.325)	0.494
		Total		40.724 (31.750–52.236)	49.816 (33.049–75.091)	0.424
	Hair (ppm)			0.154 (0.131–0.180)	0.137 (0.100–0.187)	0.484

GM (95% CI): geometric means (95% confidence limit); Organic arsenic: DMA + MMA; Inorganic arsenic: As III + As V; Total urine arsenic: DMA + MMA + As III + As V; Limit of detection: As III 0.176; As V 0.135; DMA 0.121; MMA 0.198; Adjusted for sex, age, education level, smoking status, drinking status, job, duration of residence

**Table 4:** Arsenic concentrations of the adult and child exposure groups at the first and second tests.

Arsenic Species in Urine			GM (95% CI)		AM ± Std (Pre-Post)	*p*-Value
			1st (Pre)	2nd (Post)		
Adult (*n* = 74)	Organic As	DMA	60.250 (50.703–71.596)	51.890 (43.505–61.892)	10.962 ± 68.578	0.173
		MMA	3.258 (2.292–4.630)	2.167 (1.459–3.220)	1.579 ± 3.703	<0.001
		Subtotal	64.638 (54.501–76.661)	55.532 (46.859–65.811)	12.541 ± 69.967	0.127
	Inorganic As	As III	0.130 (0.053–0.318)	0.006 (0.003–0.012)	1.269 ± 1.585	<0.001
		As V	0.594 (0.380–0.927)	0.011 (0.005–0.023)	0.926 ± 0.893	<0.001
		Subtotal	1.897 (1.389–2.591)	0.034 (0.015–0.079)	2.195 ± 1.897	<0.001
	Total		67.348 (56.846–79.789)	56.356 (47.569–66.767)	14.736 ± 70.798	0.078
Child (*n* = 45)	Organic As	DMA	37.160 (29.820–46.305)	40.781 (32.260–51.554)	−6.291 ± 44.580	0.349
		MMA	1.738 (0.928–3.255)	1.346 (0.715–2.534)	0.363 ± 1.927	0.212
		Subtotal	40.161 (32.361–49.841)	43.114 (34.098–54.513)	−5.928 ± 45.285	0.385
	Inorganic As	As III	0.037 (0.012–0.115)	0.002 (0.001–0.003)	0.765 ± 1.007	<0.001
		As V	1.294 (1.160–1.442)	0.006 (0.003–0.015)	1.171 ± 0.647	<0.001
		Subtotal	1.961 (1.664–2.310)	0.009 (0.003–0.023)	1.936 ± 1.210	<0.001
	Total		42.482 (34.448–52.390)	43.446 (34.405–54.862)	−3.992 ± 45.436	0.559

GM (95% CI): geometric means (95% confidence limit); AM ± Std: Arithmetic means ± standard deviation; Organic arsenic: DMA + MMA; Inorganic arsenic: As III + As V; Total urine arsenic: DMA + MMA + As III + As V; Limit of detection: As III 0.176; As V 0.135; DMA 0.121; MMA 0.198

**Table 5:** Comparison of the diagnostic test results between the adult exposure and control groups.

Diagnostic Test		AM ± Std		*p*-Value
		Exposure (*n* = 99)	Control (*n* = 64)	
Blood pressure	Systolic	121.26 ± 15.20	132.42 ± 16.47	<0.001
	Diastolic	70.61 ± 10.86	72.52 ± 9.23	0.247
Complete blood cell count	White blood cell	6.32 ± 1.81	7.09 ± 2.60	0.032
	Red blood cell	4.33 ± 0.44	4.69 ± 3.99	0.374
	Hemoglobin	13.11 ± 1.66	12.61 ± 1.74	0.073
	Platelet	250.84 ± 70.56	269.77 ± 73.84	0.103
Liver Function Test	Glutamic oxalacetic transaminase	26.79 ± 8.33	25.27 ± 6.12	0.210
	Glutamic pyruvate transaminase	19.91 ± 12.86	19.58 ± 9.53	0.860
	Gamma glutamyl transpeptidase	31.75 ± 29.40	30.67 ± 27.16	0.815
Renal function test	Blood urea nitrogen	17.35 ± 5.54	18.41 ± 4.51	0.205
	Creatinine	0.94 ± 0.20	0.89 ± 0.16	0.111
	Beta 2 microglobulin	0.24 ± 0.10	0.24 ± 0.09	0.969
Tumor marker	Alpha fetoprotein	2.55 ± 1.35	2.69 ± 1.49	0.521
	Carcinoembryonic antigen	2.79 ± 2.49	2.55 ± 1.10	0.398
	Carbohydrate antigen 19-9	6.79 ± 6.89	4.53 ± 3.41	0.006
	Cancer antigen 125	10.80 ± 4.93	10.34 ± 4.30	0.601
	Prostate specific antigen	1.68 ± 1.91	1.95 ± 2.23	0.688
	cytokeratin-19 fragments	1.45 ± 0.57	1.45 ± 1.03	0.956

AM ± Std: Arithmetic means ± standard deviation; Reference value: Alpha fetoprotein < 10 ng/mL; Carcinoembryonic antigen < 4.7 ng/mL; Carbohydrate antigen 19-9 < 37 U/mL; Cancer antigen 125 < 37 U/mL; Prostate specific antigen < 4.1 ng/mL; cytokeratin-19 fragments < 3.3 ng/mL

**Table 6:** Arsenic concentration in drinking water from the villages.

Name of Town	Name of Village	Source	Concentration of As (mg/L)		
			Before the Installation of Filter		After The Installation of Filter
			1st	2nd	
A	a	ground water	0.0253	0.0260	<0.0015
B	b	ground water	0.0232	0.0241	<0.0015
C	c	ground water	0.0176	0.0155	<0.0015
C	d	Ground water and stream water	0.1040	0.0972	<0.0015
C	e	ground water	0.0342	0.0286	<0.0015

Korean potable drinking water standard: 0.01 mg/L; Limit of detection of water: 0.0015 mg/L

**Table 7:** Arsenic concentration in the soil and rice crop specimens collected from the studied area.

Name of Town	Name of Village	Average Concentration of As in Soils (mg/kg)		Average Concentration of As of Rice (mg/kg)	
		*n*	AM ± Std	*n*	AM ± Std
A	a	28	12.531 ± 3.098	15	0.155 ± 0.082
B	b	12	9.472 ± 2.009	12	0.173 ± 0.091
C	c	20	16.367 ± 8.766	19	0.212 ± 0.169
C	e	30	13.835 ± 6.531	7	0.031 ± 0.026
C	f	14	5.274 ± 1.814	22	0.118 ± 0.042
C	g	10	7.142 ± 2.180	10	0.087 ± 0.040

Korean environmental standard for As in soil: 25 mg/kg; Korean environmental standard for As in rice: 0.2 mg/kg; Limit of detection of soil: 0.002 mg/kg; Limit of detection of rice: 0.0075 mg/kg; AM ± Std: Arithmetic means ± standard deviation

## References

[1] Mandal B.K., Suzuki K.T. (2002). Arsenic round the world: A review. Talanta.

[2] Guo X.J., Fujino Y., Kaneko S., Wu K., Xia Y., Yoshimura T. (2001). Arsenic contamination of groundwater and prevalence of arsenical dermatosis in the hetao plain area, Inner Mongolia, China. Molecular Mechanisms of Metal Toxicity and Carcinogenesis.

[3] Milton A.H., Rahman M. (2002). Respiratory effects and arsenic contaminated well water in Bangladesh. Int. J. Environ. Health Res..

[4] Rodrıguez V., Jimenez-Capdeville M., Giordano M. (2003). The effects of arsenic exposure on the nervous system. Toxicol. Lett..

[5] Ahmad S.A., Sayed M., Barua S., Khan M.H., Faruquee M., Jalil A., Hadi S.A., Talukder H.K. (2001). Arsenic in drinking water and pregnancy outcomes. Environ. Health Perspect..

[6] Zelikoff J.T., Bertin J.E., Burbacher T.M., Hunter E.S., Miller R.K., Silbergeld E.K., Tabacova S., Rogers J.M. (1995). Health risks associated with prenatal metal exposure. Toxicol. Sci..

[7] Mazumder D.G. (2005). Effect of chronic intake of arsenic-contaminated water on liver. Toxicol. Appl. Pharmacol..

[8] Tsai S.-Y., Chou H.-Y., The H.-W., Chen C.-M., Chen C.-J. (2003). The effects of chronic arsenic exposure from drinking water on the neurobehavioral development in adolescence. Neurotoxicology.

[9] Wasserman G.A., Liu X., Parvez F., Ahsan H., Factor-Litvak P., van Geen A., Slavkovich V., Lolacono N.J., Cheng Z., Hussain I. (2004). Water arsenic exposure and children’s intellectual function in Araihazar, Bangladesh. Environ. Health Perspect..

[10] Lai M.-S., Hsueh Y.-M., Chen C.-J., Shyu M.-P., Chen S.-Y., Kuo T.-L., Wu M.-M., Tai T.-Y. (1994). Ingested inorganic arsenic and prevalence of diabetes mellitus. Am. J. Epidemiol..

[11] Tseng C.-H., Tseng C.-P., Chiou H.-Y., Hsueh Y.-M., Chong C.-K., Chen C.-J. (2002). Epidemiologic evidence of diabetogenic effect of arsenic. Toxicol. Lett..

[12] Ahsan H., Perrin M., Rahman A., Parvez F., Stute M., Zheng Y., Milton A.H., Brandt-Rauf P., Van Geen A., Graziano J. (2000). Associations between drinking water and urinary arsenic levels and skin lesions in Bangladesh. J. Occup. Environ. Med..

[13] Knobeloch L.M., Zierold K.M., Anderson H.A. (2006). Association of arsenic-contaminated drinking-water with prevalence of skin cancer in Wisconsin’s Fox River Valley. J. Health Popul. Nutr..

[14] US Environmental Protection Agency Drinking Water Standards and Health Advisory Tables. https://www.epa.gov/dwstandardsregulations/drinking-water-standards-and-health-advisory-tables.

[15] US Food Drug Administration, Health & Human Services (2009). Beverages: Bottled water (Final rule). Federal Regist..

[16] Korea Ministry of Environment Standard of Drinking Water. https://www.me.go.kr/home/web/policy_data/read.do;jsessionid=Vsffm2wnIlOrybDL1y5pxU1anACSAeOsZZIa6uHWv1wcqX5dguGJmv2XBYFjRPsm.meweb2vhost_servlet_engine1?pagerOffset=40&maxPageItems=10&maxIndexPages=10&searchKey=&searchValue=&menuId=10264&orgCd=&condition.code=A5&condition.deleteYn=N&seq=6602.

[17] American Public Health Association, American Water Works Association, Water Environment Federation (2005). Standard Methods for the Examination of Water and Wastewater.

[18] US Environmental Protection Agency (1994). Methods for Chemical Analysis of Water and Wastes, T. Method 200.8, Determination of Trace Elements and Wastes by Inductively Coupled Plasma-Mass Spectrometry.

[19] US Environmental Protection Agency (1995). Test Methods for Evaluating Solid Waste, E. Method 3051, Microwave Assisted Acid Digestion of Sediments, Sludges, Soils, and Oils.

[20] Kurokawa M., Ogata K., Idemori M., Tsumori S., Miyaguni H., Inoue S., Hotta N. (2001). Investigation of skin manifestations of arsenicism due to intake ofarsenic-contaminated groundwater in residents of Samta, Jessore, Bangladesh. Arch. Dermatol..

[21] Smith A.H., Goycolea M., Haque R., Biggs M.L. (1998). Marked increase in bladder and lung cancer mortality in a region of northern Chile due to arsenic in drinking water. Am. J. Epidemiol..

[22] Hopenhayn-Rich C., Biggs M.L., Smith A.H. (1998). Lung and kidney cancer mortality associated with arsenic in drinking water in Cordoba, Argentina. Int. J. Epidemiol..

[23] Ferreccio C., González C., Milosavjlevic V., Marshall G., Sancha A.M., Smith A.H. (2000). Lung cancer and arsenic concentrations in drinking water in Chile. Epidemiology.

[24] Yoshida T., Yamauchi H., Sun G.F. (2004). Chronic health effects in people exposed to arsenic via the drinking water: Dose–response relationships in review. Toxicol. Appl. Pharmacol..

[25] Brown K.G., Boyle K.E., Chen C.W., Gibb H.J. (1989). A dose-response analysis of skin cancer from inorganic arsenic in drinking water. Risk Anal..

[26] Abernathy C., Marcus W., Chen C., Gibb H., White P., Cork P., Preuss P. (1998). Report on Arsenic Work Group Meeting.

[27] Tseng W., Chu H.M., How S., Fong J., Lin C., Yeh S. (1968). Prevalence of skin cancer in an endemic area of chronic arsenicism in Taiwan 2. J. Nat. Cancer Inst..

[28] Tseng W.-P. (1977). Effects and dose-response relationships of skin cancer and Blackfoot disease with arsenic. Environ. Health Perspect..

[29] Smith A.H., Hopenhayn-Rich C., Bates M.N., Goeden H.M., Hertz-Picciotto I., Duggan H.M., Wood R., Kosnett M.J., Smith M.T. (1992). Cancer risks from arsenic in drinking water. Environ. Health Perspect..

[30] Li Z. (2018). Health risk characterization of maximum legal exposures for persistent organic pollutant (POP) pesticides in residential soil: An analysis. J. Environ. Manag..

[31] Li Z., Jennings A.A. (2017). Implied maximum dose analysis of standard values of 25 pesticides based on major human exposure pathways. AIMS Public Health.

[32] Pandey P.K., Yadav S., Pandey M. (2007). Human arsenic poisoning issues in central-east Indian locations: Biomarkers and biochemical monitoring. Int. J. Environ. Res. Public Health.

[33] Susko M.L., Bloom M.S., Neamtiu I.A., Appleton A.A., Surdu S., Pop C., Fitzgerald E.F., Anastasiu D., Gurzau E.S. (2017). Low-level arsenic exposure via drinking water consumption and female fecundity—A preliminary investigation. Environ. Res..

[34] Yu Y., Guo Y., Zhang J., Xie J., Zhu Y., Yan J., Wang B., Li Z. (2017). A perspective of chronic low exposure of arsenic on non-working women: Risk of hypertension. Sci. Total Environ..

[35] Park S.K., Peng Q., Bielak L.F., Silver K.D., Peyser P.A., Mitchell B.D. (2016). Arsenic exposure is associated with diminished insulin sensitivity in non-diabetic Amish adults. Diabetes-Metab. Res. Rev..

[36] Krohn R.M., Lemaire M., Silva L.F.N., Lemarié C., Bolt A., Mann K.K., Smits J.E. (2016). High-selenium lentil diet protects against arsenic-induced atherosclerosis in a mouse model. J. Nutr. Biochem..

